# Evaluation and *In Situ* Library Expansion of Small Molecule MHC-I Inducers

**DOI:** 10.1101/2025.01.31.635109

**Published:** 2025-02-05

**Authors:** Joey J. Kelly, Sarah E. Newkirk, Mahendra D. Chordia, Marcos M. Pires

**Affiliations:** 1 Department of Chemistry, University of Virginia, Charlottesville, VA, United States 22904; 2 Department of Microbiology, Immunology, and Cancer, University of Virginia, Charlottesville, VA, United States 22904

## Abstract

Immunotherapy has emerged as a powerful strategy for combating cancer by harnessing the patient immune system to recognize and eliminate malignant cells. The major histocompatibility complex class I (MHC-I) has a pivotal role in the recognition step. These surface proteins present cancer-specific neoantigens to CD8+ T cells, which triggers activation and T cell-mediated killing. However, cancer cells can often evade immune detection by downregulating MHC-I surface expression, which renders the immune response less effective. In turn, this resistance mechanism offers an opportunity to bolster MHC-I surface expression *via* therapeutic interventions. Here, we conducted an initial comprehensive evaluation of previously purported small molecule MHC-I inducers and identified heat shock protein 90 (Hsp90) inhibitors as privileged inducers of MHC-I surface expression. With a core scaffold in hand, we employed an *in situ* click chemistry-based derivatization strategy to generate 380 novel compounds in the same family. New agents from this library showed high levels of induction, with one of the triazole-based analogs, **CliMB-325**, also enhancing T cell activation and exhibiting lower toxicity, which could potentiate some immunotherapeutic modalities. Moreover, we demonstrated the potential of a click chemistry-based diversification strategy for the discovery of small molecules to counter immune evasion.

## INTRODUCTION

The immune system can, often times, be precise, efficient, and powerful in detecting and eliminating cancerous cells. One of the principal mechanisms that the immune system leverages for cancer cell detection is through the presentation of cancer-specific peptides on the major histocompatibility complex (MHC) of the pathogenic cell.^1^ In particular, MHC class I (MHC-I) is a membrane protein expressed on most nucleated cells and is responsible for presenting short (typically 8–12 amino acids long) peptides to CD8+ T cells.^2^ Recognition of a peptide-MHC complex (pMHC) by a CD8+ T cell through its T cell receptor (TCR) can result in a cytotoxic response through release of a host of agents including perforin and granzyme B.^3^ For a CD8+ T cell to be activated against a target cell (and undergo subsequent phenotypic changes), it must first recognize a ‘non-self’ peptide which are generated inside the cell and presented on MHC-I. The types of non-self peptides that are typically found on the surface cancer cells are broadly known as neoantigens.^4^

**Figure 1.**
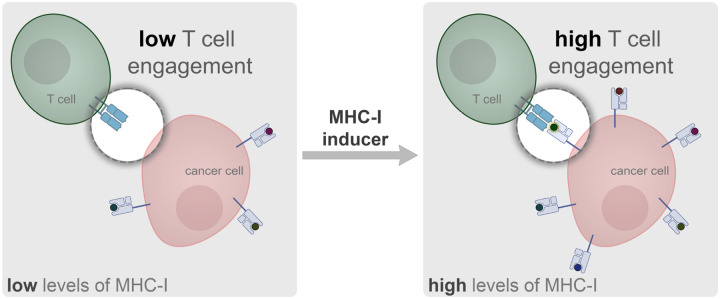
Schematic representation of strategy to increase MHC-I expression and CD8+ T cell response. Cancer cells can downregulate the expression of MHC-I as a mechanism of evasion from a patient’s immunosurveillance. The use of small molecule inducers could potentially enhance immunotherapeutic approaches that are widely used in the clinic.

Neoantigens are generated *via* structural alterations to the proteome of cancer cells through amino acid substitution,^5^ post-translational modifications,^6^ and other mechanisms.^7^ These non-self peptides, which can be loaded on MHC-I for presentation, can potentially engage with TCRs on T cells.^8^ Therefore, CD8+ T cells displaying the cognate TCR are well positioned to specifically recognize and respond to neoantigen-presenting cancer cells to promote an anti-cancer immune response.^7^ In many instances, these mechanisms are central to eliminate the emergence of cancerous cells. Yet, there is considerable evidence demonstrating that cancer cells can actively avoid immune recognition by CD8+ T cells.^9, 10^ These mechanisms of resistance include, but are not limited to, remodeling of the tumor environment to be hypoxic and immunosuppressive,^11–13^ increasing expression of immune checkpoint proteins (e.g., programmed death-ligand (PD-L1) and cytotoxic T-lymphocyte associated protein 4 (CTLA-4),^14, 15^ promoting the secretion of immunosuppressive cytokines,^16^ and downregulating MHC-I molecules.^17^ Critically, the downregulation of surface MHC-I can directly impair patient response to programmed cell death protein 1 (PD-1) blockade immunotherapy.^18^ Given the tremendous success of cancer immunotherapy that directly relies on MHC-I/TCR engagement, there is a clear need to discover potent agents that promote the expression of MHC-I in cancer patients. By restoring or enhancing MHC-I expression, it may be possible to overcome immune resistance observed in many cancers, thus making the cancer cell more susceptible to T cell-mediated killing (Figure 1).

The concept of using small molecules to promote the expression of MHC-I is not novel in and of itself. There have been a number of reports that identified compounds with this purported function. These compounds span a diverse set of biological functions and include DNA methyltransferase (DMNT) inhibitors,^19–26^ histone deacetylase (HDAC) inhibitors,^27–29^ kinase inhibitors,^30^ heat shock protein 90 (Hsp90) inhibitors,^31, 32^ stimulator of interferon genes (STING) agonists,^33–35^ and others.^36–39^ Although a wider range of FDA approved agents have been screened for MHC-I induction,^40, 41^ principally, to the best of our knowledge, a definitive comparison across small molecule inducers has not been previously reported. Given the diversity of reagents across the prior reports (e.g., cell lines, antibodies, concentrations, incubation times, etc.), it is critical to first establish the best-in-class scaffold. In this work, we conducted a rigorous head-to-head screen of small molecules to compare their ability to enhance MHC-I surface expression in colorectal cancer cells. Additionally, we show that Hsp90 inhibitors can increase the presentation of cancer-specific neoantigens. With a privileged scaffold in hand, we conducted a high-throughput diversification screen to generate a range of analogs that could be evaluated for their pharmacological properties.

## RESULTS

### Screening Small Molecules for their MHC-I Upregulation Activity

**Figure 2.**
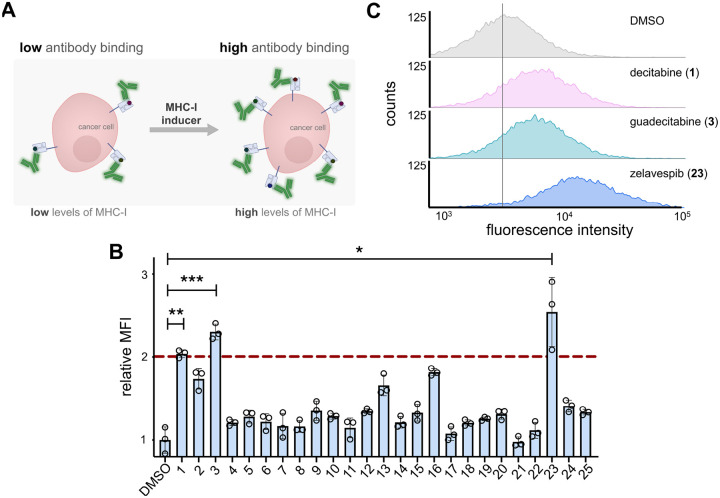
**(A)** Schematic representation of fluorescent antibody readout for increased MHC-I surface expression upon treatment with small molecules inducers. **(B)** Flow cytometry analysis of CT26 cells treated with 1 μM of indicated compounds (corresponding names and structures found in Figure S1). Red dashed lined indicates threshold of 2-fold increase in MHC-I surface expression relative to DMSO control. MFI is the mean fluorescence intensity of the level of fluorescence relative to the DMSO control. Data are represented as mean ± SD (n=3). p-values were determined by a two-tailed *t*-test (* p < 0.05, ** p < 0.01, *** p < 0.001). **(C)** Flow cytometry histograms of CT26 cells incubated with 500 nM of indicated compounds. H-2K^d^ expression was measured by APC anti-mouse H-2K^d^ antibody. The vertical grey line represents median fluorescence intensity of DMSO treated cells.

To identify the best-in-class drug scaffold for MHC-I upregulation, a flow cytometry-based assay was developed. Briefly, CT26 murine colorectal cancer cells were incubated with individual members of a library of 25 small molecules that have previously been shown to increase MHC-I expression. These included DNMT, kinase, HDAC, bromodomain and extra-terminal (BET), proteasome, and Hsp90 inhibitors, as well as STING agonists and IMiDs (Figure S1). After cellular treatment with each individual compound, a fluorescent anti-H-2K^d^ antibody was used to quantify MHC-I expression *via* flow cytometry (Figure 2A). As expected at this high concentration, most of these molecules showed an increase in MHC-I surface expression at a concentration of 5 μM (Figure S2). Still, it was notable that some of the molecules did not show any enhancement above background. To identify the most potent inducers of MHC-I surface expression, a second screen was performed at a more stringent concentration of 1 μM. Our results showed that three of the small molecules exhibited an increase in MHC-I surface expression above a two-fold cutoff (Figure 2B). The identified MHC-I enhancers were primarily in two pharmacological classes: DNMT inhibitors (decitabine **1** and guadecitabine **3**) and Hsp90 inhibitors (zelavespib **23**). After incubation of the three lead compounds at a concentration of 500 nM with CT26 cells, it was found that **23** led to higher overall expression of MHC-I on the surface compared to the two DNMT inhibitors (Figure 2C). Therefore, we decided to focus on Hsp90 inhibitors and expand our search within this class beyond zelavespib in search of the most potent MHC-I inducers.

### Screening Hsp90 Inhibitors for MHC-I Surface Upregulation Activity

**Figure 3.**
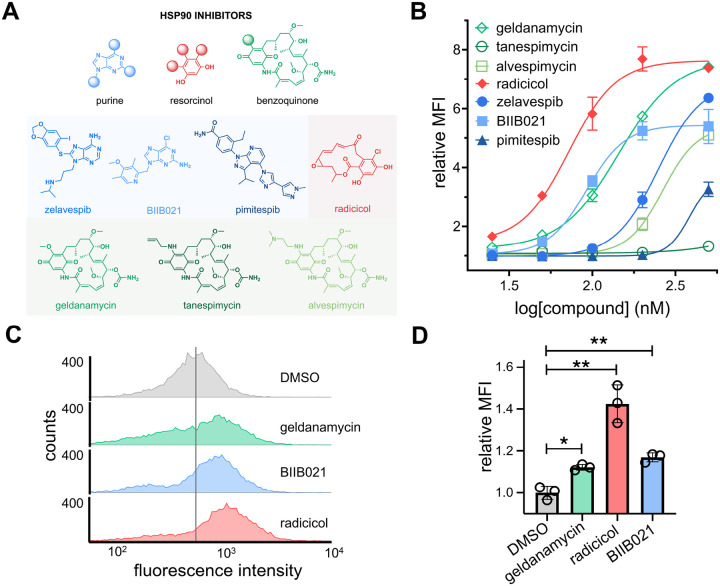
**(A)** Chemical structures of seven Hsp90 inhibitors tested for their enhancement of MHC-I surface expression. **(B)** Dose-response analysis by flow cytometry of CT26 cells treated with varying concentrations of seven Hsp90 inhibitors. H-2K^d^ expression was measured by APC anti-mouse H-2K^d^ antibody. Data are represented as mean ± SD (n=3), and Boltzmann sigmoidal curves were fitted to the data using GraphPad Prism. EC_50_ values are the concentration of compound needed to achieve 50% of the maximal MHC-I surface expression levels. **(C)** Flow cytometry histograms of HCT116 cells incubated with 200 nM of indicated compounds. HLA-A, B, C surface expression was measured by APC anti-human HLA-A, B, C antibody. The vertical grey line represents median fluorescence intensity of DMSO treated cells. **(D)** Flow cytometry analysis of MC38-OVA cells treated with 100 nM of indicated compound. SIINFEKL-H2-K^b^ expression was measured by APC anti-mouse H-2K^b^ bound to SIINFEKL antibody. MFI is the mean fluorescence intensity of the level of fluorescence relative to the DMSO control. Data are represented as mean ± SD (n=3). p-values were determined by a two-tailed *t*-test (* p < 0.05, ** p < 0.01, **** p < 0.0001).

To further explore the relationship between Hsp90 inhibitors and MHC-I surface expression, six additional Hsp90 inhibitors were examined (Figure 3A). In total within this sub-library, three of the molecules were from the purine-based family, one from the resorcinol family, and three from the benzoquinone family of Hsp90 inhibitors. To more readily assess their potency, a concentration scan was performed in CT26 cells rather than a single concentration analysis. Our results revealed that all but two (pimitespib and tanespimycin) of the Hsp90 inhibitors tested had EC_50_ values in the nanomolar range for MHC-I surface expression. Among these, it was found that radicicol, BIIB021 and geldanamycin had the lowest EC_50_ values, at 72, 92, and 144 nM, respectively. Interestingly, together these top hits covered all three primary classes of Hsp90 inhibitors; we pose that this could suggest that Hsp90 inhibition is a primary driver of the phenotypic observation of MHC-I induction. Presumably, if any off-target activity were to be observed and if it were to be the driver of the induction, there is likely to be a single class that is favored. We note that the top hits enhanced MHC-I surface expression in CT26 cells by approximately 5 to 8-fold compared to basal expression levels, a marked increase relative to the initial hit that prompted the focus on Hsp90 inhibitors (Figure 3B). To ensure that Hsp90 inhibitors could operate in other cellular contexts, we tested them in the human colorectal cancer cell line HCT116 and found them to be effective MHC-I inducers (Figure 3C). Overall, these results demonstrate that Hsp90 inhibitors are potent inducers of MHC-I surface expression, and this prompted us to further explore them in assembling a larger and more diverse structure activity relationship campaign.

In theory, the enhancement of MHC-I surface expression should broadly sample a greater breadth of cytosolic peptides including potential neoantigens. This is key because the efficacy of checkpoint blockage therapy relies principally on cytotoxic CD8+ T cells recognizing neoantigens presented on cancer cell surfaces. We next investigated the potential upregulation of specific antigens from live cells upon their treatment with Hsp90 inhibitors (Figure 3D). To test this, we used murine MC38-OVA cells which are genetically modified to express the protein ovalbumin (OVA).^42^ OVA contains the sequence SIINFEKL, which has been previously used as a model neoantigen. Upon the intracellular processing of OVA and the production of SIINFEKL, it is known that this peptide can be presented by H-2K^b^ and is recognized by SIINFEKL-specific CD8+ T cells.^43^ MC38-OVA cells were treated with 100 nM of Hsp90 inhibitors for 48 hours, followed by incubation with a fluorescent antibody specific for H-2K^b^ bound to SIINFEKL. Satisfyingly, cellular treatment with Hsp90 inhibitors led to a significant increase in the presentation of the model neoantigen SIINFEKL, indicating that Hsp90 inhibitors can potentially promote the presentation of neoantigen-specific pMHC complexes (Figure 3D).

### High-Throughput Click Chemistry Diversification Strategy of Hsp90 Inhibitor

With the three top candidates in hand, we sought to further diversify a core scaffold to broadly understand how structure could potentially drive MHC-I upregulation. We posed that a large-scale sub-library around a single agent could provide us with a larger set of agents that can be tested and selected for specific biological properties (e.g., improved toxicity profile, solubility, and selectivity). From the three agents, we moved forward with BIIB021 due to its potency (with many similar structures in clinical evaluation for Hsp90 inhibition)^44–46^ and its robust chemical structure. Given the nature of our derivatization strategy, it was important to consider the potential stability of the starting scaffold; both radicicol and geldanamycin have structural fragments that are known to have low inherent chemical stability. Also, the availability of the crystal structure of BIIB021 in complex with Hsp90 can provide an avenue to understand how the analogs may be interacting with their target protein.^47^

For the generation of the library, we chose to use *in situ* click chemistry. In this format, an alkyne is installed within the core scaffold, and this parent molecule is plated into a microwell plate system with each well containing a new azide-tagged fragment. This approach presents considerable advantages for drug discovery of MHC-I inducers. Click reactions have a high level of specificity and efficiency, particularly exemplified by the Cu(I)-catalyzed azide-alkyne cycloaddition (CuAAC), which enables precise modifications and synthesizes complex molecules with minimal byproducts.^48–50^ The versatility inherent in using a library of azides allows for the exploration of diverse molecular combinations and structural variations, essential for identifying drug candidates with optimal pharmacological properties. Moreover, it facilitates the simultaneous screening and synthesis of potential drug candidates, speeding up the identification of active compounds. Recently, this click chemistry-based strategy has been used to identify small molecule modulators of glucagon-like-peptide-1 receptor.^51^ It has also been shown with this method that over 80% of azide molecules formed triazole products at yields of 70% or higher.^52^

**Figure 4.**
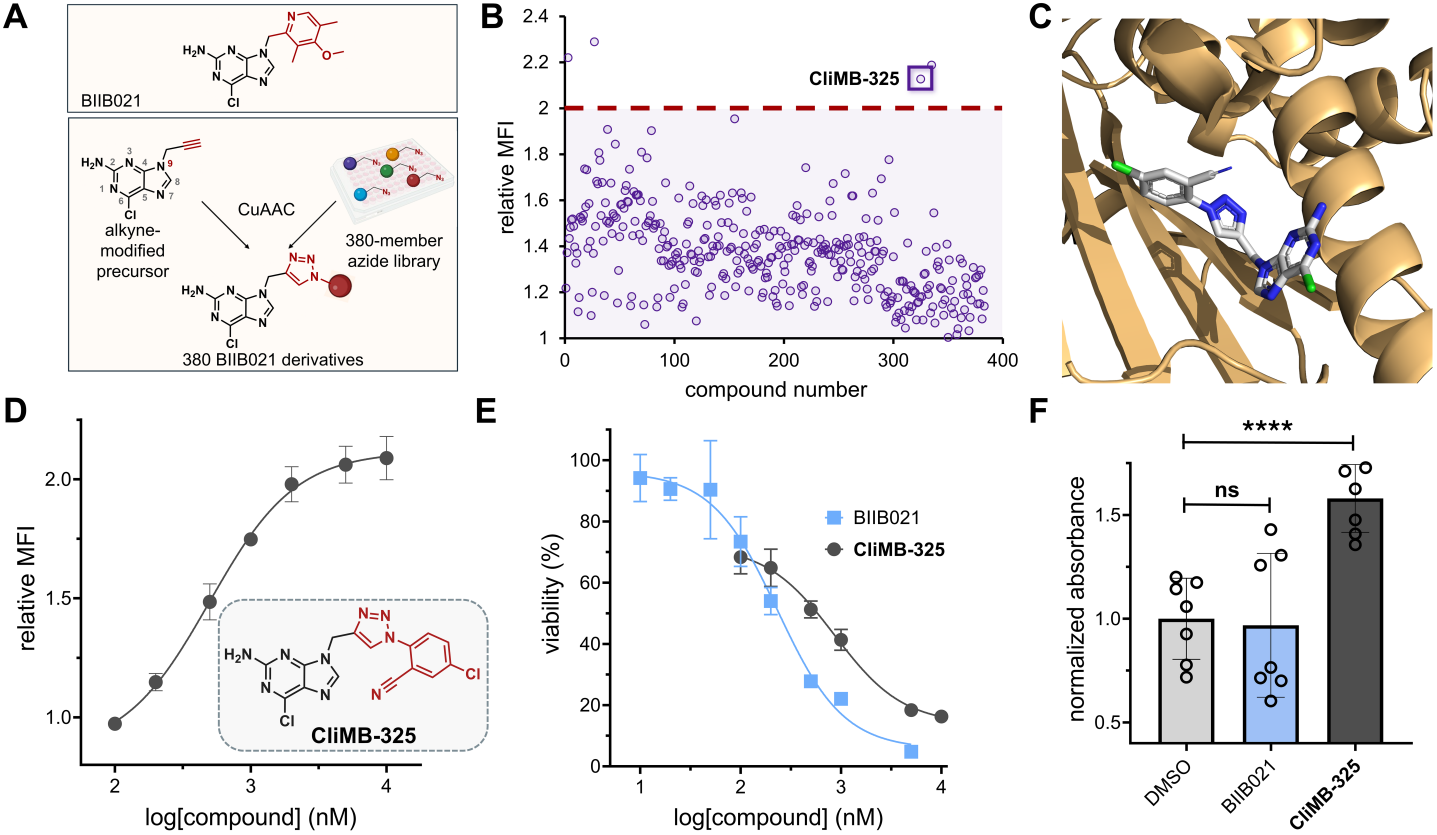
**(A)** Schematic representation of BIIB021 derivatization strategy. **(B)** Flow cytometry analysis of CT26 cells treated with click products between 9-propargyl-2-amino-6-chloropurine and the 380-member azide library. H-2K^d^ expression was measured by APC anti-mouse H-2K^d^ antibody and performed in singlet. Red dashed lined indicates threshold of 2-fold increase in MHC-I surface expression relative to DMSO control (corresponding structures of azides found in Figure S8). MFI is the mean fluorescence intensity of the level of fluorescence relative to the DMSO control. **(C)** Stick model of **CliMB-325** (white) docked into Hsp90 (light orange) was generated using existing crystal structure data (PDB ID: 3qdd) and Rosetta. **(D)** Dose-response curve and chemical structure of **CliMB-325**. CT26 cells were treated with varying concentrations of **CliMB-325**. H-2K^d^ expression was measured by APC anti-mouse H-2K^d^ antibody via flow cytometry. MFI is the mean fluorescence intensity of the level of fluorescence relative to the DMSO control. Data are represented as mean ± SD (n=3), and Boltzmann sigmoidal curves were fitted to the data using GraphPad Prism. EC_50_ values are the concentration of compound needed to achieve 50% of the maximal MHC-I surface expression levels. **(E)** Dose-response curves of CT26 cells treated with varying concentrations of **CliMB-325** or BIIB021 determined via MTT cell viability assay. Data are represented as mean ± SD (n=4), and nonlinear regression curves were fitted to the data using GraphPad Prism. IC50 values are the concentration of compound at which maximal cell viability is inhibited by 50%. **(F)** MC38-OVA cells were incubated with 100 nM BIIB021 and 1 μM **CliMB-325** for 48 hours. Subsequently, cells were co-cultured with B3Z T cells for six hours. β-galactosidase expression was then measured via the colorimetric reagent CPRG on a plate reader at 570 nm. Data are represented as mean ± SD (n=7). p-values were determined by a two-tailed *t*-test (ns = not significant, **** p < 0.0001).

In the context of our core purine scaffold, we needed to consider a site to install the alkyne handle. From the crystal structure of BIIB021 in complex with Hsp90, we identified the N9 position of the purine core as a solvent-exposed site that is amendable to chemical modification.^47^ Moreover, previous reports have demonstrated that this position can be leveraged to access purine-based analogs while retaining Hsp90 inhibition.^53^ Therefore, our envisioned approach involved modifying a precursor of BIIB021, 2-amino-6-chloropurine, with an alkyne on the N9 position in order to react it with a library of small molecule azides (Figure 4A). We reasoned that the resulting triazole ring from the click reaction would structurally mimic the pyridine ring of BIIB021 and allow us to rapidly generate hundreds of derivatives.

To build the alkyne-bearing purine, we reacted 2-amino-6-chloropurine with propargyl bromide to yield the major product 9-propargyl-2-amino-6-chloropurine. The other product was the N7 regioisomer, which was separated during purification. The identity of isolated N9 alkyne-bearing compound was confirmed by NMR and its purity was analyzed by RP-HPLC. Next, a model *in situ* CuAAC test reaction was performed with the alkyne-modified precursor and a small subset of azide bearing molecules. To cover the potential variability in types of chemical structures found in the full library, we selected a subset of molecules that varied in size, polarity, and steric environment surrounding the azide. Of note, the reactions were performed in microwell plates in an experimental procedure that mimicked the conditions for the eventual *in situ* set of reactions. All three reactions showed a conversion rate of >90% to the triazole product (Figure S4–S6). With the model reactions showing high levels of conversion, we reasoned that the reaction conditions were wellsuited for a larger screen. The goal of utilizing a larger library was to ensure that modifications to the purine core would cover a large chemical space to broadly sample the engagement with the target. Moreover, given the nature of the phenotypic assay, we reasoned that the diversity of this library could also be important in improving other properties that are necessary for a lead candidate including high accumulation levels, low off-target effects, and reduced toxicity.

In total, 380 azide-containing small molecules were dispensed into wells containing the alkyne-bearing purine analog and the click reaction reagents. After the reaction step, contents of each well were incubated with CT26 cells, and MHC-I induction was monitored by treatment with a fluorescently tagged antibody as previously described. Critically, incubation of CT26 cells with the alkyne precursor alone or the additional click reagents did not result in any increase in MHC-I surface expression (Figure S7). This indicates that the reaction reagents have no activity on their own. The results from the 380-member screen revealed that four of the click reaction mixtures (3, 27, 325, and 335; structures shown in Figure S8), showed an increase in MHC-I surface expression above a two-fold cutoff over the DMSO control (Figure 4B). To further confirm these results, cells were treated with each of four reaction mixtures in a more stringent concentration (theoretically 500 nM, assuming complete conversion). From these results, cell treatment with compound 325 led to the highest levels of MHC-I surface expression (Figure S9). The click product between the alkyne precursor and compound 325 of the azide library was then synthesized and purified to yield ‘**Cli**ck **M**HC-I **B**ooster-**325** (**CliMB-325**).

### CliMB-325 Enhances MHC-I Surface Expression and T Cell Activation

Finally, we sought to further characterize our novel purine-based lead agent **CliMB-325**. By docking **CliMB-325** into Hsp90 using RosettaLigand, we found that **CliMB-325** binds in a similar manner to BIIB021 (PDB ID: 3qdd) with no measurable deviation in Hsp90 conformation (Figure 4C). Next, to establish its potency, a concentration scan of **CliMB-325** was performed using CT26 cells to assess its ability to increase MHC-I expression. Our results showed that **CliMB-325** retained MHC-I upregulation activity with an EC_50_ of 498 nM (Figure 4D). Given the failed clinical progression of BIIB021 due to toxicity issues,^44^ we also performed an MTT cell viability assay comparing BIIB021 with **CliMB-325**. We found that **CliMB-325** demonstrated a 3.7-fold improvement in toxicity over its parent compound, BIIB021, with IC_50_ values of 861 nM and 233 nM, respectively (Figure 4E).

Given that the downregulation of MHC-I surface expression reduces T cell recognition of neoantigens, we then sought to investigate whether the upregulation of MHC-I induced by **CliMB-325** results in enhanced T cell activation. To do so, we used the B3Z T cell hybridoma cell line, which contains OVA-specific TCRs and expresses the enzyme β-galactosidase under the control of an IL-2 inducible promoter. Upon B3Z recognition of the OVA-pMHC complex on OVA expressing cells, the subsequent IL-2 production promotes the expression of β-galactosidase. b-galactosidase activity can then be measured *via* hydrolysis of the reagent chlorophenol red-β-galactopyranoside (CPRG) which leads to a color change that is reflective of T cell activation levels.^54^ MC38-OVA cells were incubated with BIIB021 and **CliMB-325** for 48 hours before being co-cultured with B3Z T cells. While TCR activation in cells treated with BIIB021 did not significantly differ from the DMSO control, MC38-OVA cells treated with **CliMB-325** exhibited a nearly 1.6-fold increase in TCR activation levels (Figure 4F). Overall, these results validate the use of a high-throughput click chemistry screen to generate bioactive compounds with MHC-I upregulation activity.

## CONCLUSION

Here, we have identified compounds that have immunomodulatory effects on colorectal cancer cell lines. Hsp90 inhibitors were found to be among the most potent class of molecules tested that increase MHC-I surface expression as well as promote the display of cancer-specific neoantigens for CD8+ T cell recognition. Leveraging an Hsp90 inhibitor core scaffold, we have also demonstrated as proof-of-concept a novel high-throughput click chemistry-based screening platform for the discovery of molecules with immunomodulatory activity. While our initial screen utilized a 380-member azide library, we plan to expand to a 1,200-member azide library to sample a wider chemical space for molecules that upregulate MHC-I. Additionally, beyond the purine-based scaffold used in this study, we intend to extend this strategy to resorcinol and benzoquinone-based scaffolds, which have also been favored for the development of new Hsp90 inhibitors.^55–60^ Ultimately, however, we envision that our approach of modifying preexisting chemical scaffolds with alkynes for large-scale click chemistry-based derivatization can be broadly applicable for various phenotypic screens beyond MHC-I upregulation.

Counteracting cancer immune evasion mechanisms is necessary to enhance the efficacy of immunotherapy treatments. To this point, a significant portion of patients fail to respond to PD-1 blockade therapy due to the development of resistant tumors resulting from MHC-I downregulation.^18^ This challenge underscores the need to reengage the immune system by converting immunologically ‘cold’ tumors back into ‘hot’ tumors that can be recognized and targeted for elimination. Therefore, we anticipate that developing a widely applicable approach to increase MHC-I surface expression is a promising avenue to combat resistant cancer.

## Supplementary Material

Supplement 1
